# Detection and Quantification of Immunoregulatory miRNAs in Human Milk and Infant Milk Formula

**DOI:** 10.3390/biotech11020011

**Published:** 2022-04-20

**Authors:** Juan Manuel Vélez-Ixta, Tizziani Benítez-Guerrero, Arlene Aguilera-Hernández, Helga Martínez-Corona, Karina Corona-Cervantes, Carmen Josefina Juárez-Castelán, Martín Noé Rangel-Calvillo, Jaime García-Mena

**Affiliations:** 1 Departamento de Genética y Biología Molecular, Centro de Investigación y de Estudios Avanzados del Instituto Politécnico Nacional (Cinvestav), Av. Instituto Politécnico Nacional 2508, Ciudad de México 07360, Mexico; juan.velez@cinvestav.mx (J.M.V.-I.); tizziani.benitez@cinvestav.mx (T.B.-G.); arlene.aguilera@cinvestav.mx (A.A.-H.); helga.martinez@cinvestav.mx (H.M.-C.); karina.corona@cinvestav.mx (K.C.-C.); carmen.juarez@cinvestav.mx (C.J.J.-C.); 2 Hospital General “Dr. José María Rodríguez”, Instituto de Salud del Estado de Mexico, Ecatepec de Morelos 55200, Estado de México, Mexico; drrangelcalvillo@gmail.com

**Keywords:** human milk, qPCR, microRNA, RNA, formula milk, breastfeeding

## Abstract

Mammary gland secretory cells produce miRNA-rich milk. In humans, these miRNAs reach infant/neonate bloodstream, playing diverse roles, like neural system development, metabolism, and immune system maturation. Notwithstanding, still few works explore human milk miRNA content, and there are no reports at the population level. Our hypothesis was that miR-146b-5p, miR148a-3p, miR155-5p, mir181a-5p, and mir200a-3p immunoregulatory miRNAs are expressed in human colostrum/milk at a higher level than infant milk formulae. The aim of this work was to evaluate the expression of the five immunoregulatory miRNAs in human milk and compare it with their expression in infant milk formula. For this purpose, miRNA relative expression was measured by qPCR in cDNA prepared from total RNA extracted from sixty human colostrum/milk samples and six different formulae. The comparative Cт method 2^−ΔCт^ using exogenous cel-miR-39 as internal control was employed, followed by statistical analysis. We found the relative expression levels of miRNAs are comparable among colostrum/milk samples, and these miRNAs are present in infant milk formulae but at very low concentrations. We conclude that the relative expression of the immunomodulatory miRNAs is comparable in all the human colostrum/milk samples and is higher than the expression in formulae.

## 1. Introduction

According to the Mexican national survey ENSANUT [1], the panorama of breastfeeding in Mexican women 12–49 years old with children up to 24 months of age is not favorable since only 28.3% of children under six months are exclusively breastfed. It is common that children under 12 months of age are fed infant formula, and just a small portion of children corresponding to 29% of the studied population was breastfed up to two years of age. Therefore, these data allow us to conclude that there is still a deficiency in breastfeeding practices in Mexico that must be tackled and managed from a collaborative perspective [2].

MicroRNAs (miRNAs) are short 19–24 nucleotides non-coding RNAs that derive from characteristic hairpin precursors. They constitute one of the more abundant classes of gene-regulatory molecules, suppressing the expression profiles of protein-coding genes at the post-transcriptional level [3,4]. miRNAs have been described in human milk (HM) as unique bioactive components. During lactogenesis, milk particles are produced by secretory epithelial cells located in the alveoli of the mammary gland and are secreted in vesicles into the lumen [5]. The vesicles carrying miRNAs are taken up by the infant during feeding. Later, they are transported and assimilated by the cells to perform their function. Colostrum has an important immune function due to its high immunoglobulins and bioactive components content [6]. In the human body, miRNAs are present in extracellular fluids like saliva, urine, plasma, and milk [7]. HM contains macronutrients, micronutrients, bioactive compounds, growth factors, and immunological factors [8].

In recent years, the study of bioactive components, for example, miRNAs, immunoglobulins, maternal cells, among others, has been of great interest. There is solid evidence of the presence of miRNAs in HM, in addition, they have been found expressed in milk of other species like goat, cow, and pig [9]. In HM, miRNAs remain stable since they are associated with extracellular vesicles, fat globules, and cellular components. There is evidence that miRNAs play an important role in establishing the neonate’s immune system and have other beneficial effects on infant health [9].

Exosome-derived miRNAs, such as miR-148a, induce cell proliferation and protein expression in normal colon epithelial cells [10]. Moreover, miR-148a promotes CD70 and LFA-1 gene overexpression through hypomethylation. These genes are associated with cell survival and T-cell activation, respectively [11]. In a study done using six-month mature HM, high content of miR-181 and miR-155 was found. These miRNAs are involved in the differentiation of B cells [12]. Examples of other highly enriched miRNAs in HM are miR-17 and miR-92, which have functions, such as regulation of monocyte development, differentiation of B cells and T cells [13]. In another report, the presence of miRNAs with important immunological functions, for example, miR-148a-3p, miR-30b-5p, miR-182-5p, and miR-200a-3p, was demonstrated in exosomes derived from HM [14]. Another group showed the presence of miR-21, miR-16, and miR-146b-5p in pre-term HM [15].

The presence of miRNAs has been established in cow’s milk based infant milk formula (IMF) by next-generation sequencing and quantitative real-time PCR. In these products, the miRNA content is generally lower than HM [7]. Since cow’s milk is the most frequently used component of infant formulas, the same group reported that around 90% of miRNAs found in human milk are also found in cow and goat’s milk. On the other hand, previous studies did not detect abundant miRNA content in IMFs [16]. The aim of this work was to explore the expression of five different immunoregulatory miRNAs in Mexican HM and compare it with the content in IMF.

## 2. Materials and Methods

### 2.1. Type of Study

Descriptive cross-sectional study of five selected miRNA expression in human milk and infant milk formula (IMF).

### 2.2. Sample

Human colostrum/milk (HCM) samples were selected from a collection of samples of a previous study made in the public hospital “Dr. José María Rodríguez” located in Ecatepec de Morelos, State of Mexico (19_36 03500N, 99_ 303600W), and kept at −70 °C until used. The protocol was approved by the Ethics Committee of the General Hospital (Project identification code: 217B560002018006) [17]. The inclusion criteria were: Healthy women between 0 to 7 postpartum days, of Mexican origin for at least two generations, who gave birth by vaginal delivery or non-elective C-section with 37–41 weeks of gestational age. The exclusion criteria were: Smoking, probiotic or alcohol consumption, being affected by diabetes, overweight, or obesity, and using antibiotics during the last trimester of pregnancy. Infant milk formulae (IMF) were selected among the most consumed products in Mexico City (Table S1). IMF were reconstituted with nuclease-free water (Sigma-Aldrich^®^, St. Louis, MO, USA, Cat. W4502-1L) following the manufacturer’s instruction.

### 2.3. Oligonucleotide Selection and Design

For this research, miR-146b-5p, miR148a-3p, miR155-5p, mir181a-5p, and mir200a-3p were selected based on literature search in PubMed portal (https://pubmed.ncbi.nlm.nih.gov/, accessed on 15 August 2020), including original and review articles from years 2000 to 2020, searching for the terms “miRNAs, human milk, milk formula and immunoregulatory miRNAs”. Raw sequences were obtained from miRBase (www.mirbase.org/, accessed on 15 August 2020). Stem-loop primers for cDNA synthesis and primers for qPCR were designed using CLC Workbench v.8.1.3 software (https://www.qiagen.com/, accessed on 15 August 2020) based on a previous report [18] (Table S2).

### 2.4. RNA Extraction

RNA was extracted from 1 mL HCM or IMF, respectively. Samples were centrifugated at max speed, for 10 min, at 4 °C in Neofuge 13R Centrifuge (HealForce^®^, Shanghai, China). Cellular pellet and lipid phase were washed with cold PBS pH 7.4. Subsequently, total RNA was extracted with *mir*Vana™ miRNA Isolation Kit (Invitrogen™, Cat. AM1560, Carlsbad, CA, USA), following manufacturer instructions. 3 µL of exogenous miRNA (33 fmol) cel-miR-39 (microRNA (cel-miR-39) Spike-In Kit, Cat. 59000, Thorold ON, Canada) were added to each sample before lysis buffer. RNA was eluted in 100 µL volume and stored at −20 °C. RNA concentration and purity were assessed with NanoDrop™ 2000 (Thermo Fisher Scientific, Cat. ND-2000, Waltham, MA, USA).

### 2.5. cDNA Synthesis

cDNA synthesis was made using 5–50 ng extracted RNA in 15 μL reaction volume, using MicroRNA Reverse Transcription Kit (Applied Biosystems™, Cat. 4366596, Waltham, MA, USA), following manufacturer’s instructions. Individual cDNA reactions were prepared for each miRNA evaluated for each sample. Reactions were placed in Thermocycler 2720 (Applied Biosystems, Waltham, MA, USA) for 30 min at 16 °C; 30 min, 42 °C; 5 min, 85 °C; and 10 min, 4 °C.

### 2.6. miRNA Quantification by qPCR

qPCR was made using 1 µL of cDNA and Maxima SYBR Green/ROX qPCR Master Mix (Thermo Scientific™, Cat. K0222, Waltham, MA, USA) reagents in a final volume of 25 µL, as described by the manufacturer. Reactions were placed on PCR plates (Applied Biosystems™, Cat. 4375816, Waltham, MA, USA) and qPCR run in StepOne™ Real-Time PCR System Thermocycler (Applied Biosystems™, Cat. 4376357, Waltham, MA, USA) according to the two-step cycling protocol: Denaturation of 95 °C for 10 min; 40 cycles of 95 °C for 15 s, 60 °C for 60 s and finally, a melt curve of 95 °C for 15 s, 60 °C for 60 s, and 95 °C for 15 s. Quantitation was made in triplicate.

### 2.7. Bioinformatics and Statistical Analyses

Data obtained from this work were analyzed using R environment [19] and Rstudio 1.4.1717 software [20]. The R-libraries employed were “readr” [21], “dplyr” [22], “scales” [23], and “tidyr” [24]. Cт were calculated by Step One Software v2.2.2 (Applied Biosystems™, Waltham, Massachusetts, USA) and exported to comma separated values (.csv) format. Afterwards, the comparative Cт method 2^−ΔCт^ [25] was used for statistical analysis. Internal control for this study was cel-miR-39. One way Analysis of Variance (ANOVA) was performed to seek differences in relative miRNA levels in HCM. Kruskal–Wallis one-way analysis of variance was carried out to seek for differences in miRNA levels at different postpartum days. Further, Mann–Whitney U test (Wilcoxon rank-sum test) was used to seek differences in relative miRNA levels between both groups (HCM and IMF). Tables were elaborated with R and exported to html for inclusion in this work using ‘sjPlot’ library [26]. Plots were elaborated with ‘ggplot2’ library [27] and exported to Joint Photographic Experts Group format (.jpg) to 600 dpi/ppi resolution.

## 3. Results

### 3.1. miRNA Relative Expression Is Comparable among the Studied Human Milk Samples

**Figure 1:**
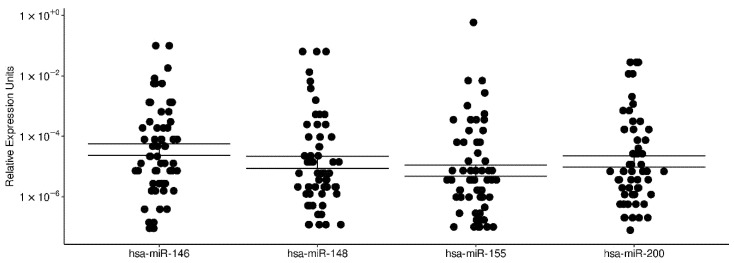
Expression of selected miRNAs in human colostrum/milk. hsa-miR146 (miR-146b-5p); hsa-miR-148 (miR148a-3p), hsa-miR-155 (miR155-5p), hsa-miR-200 (mir200a-3p). The Y-axis shows the relative expression of each miRNA with respect to the internal control cel-miR-39, and the X-axis shows the corresponding miRNA. Each dot in the plot represents a sample. The double horizontal lines indicate the standard error of the mean. One-way ANOVA statistical test shows no significant differences in miRNA levels, Pr(>F) = 0.7136.

We studied the expression of five different immuno-related miRNAs in human colostrum/milk samples collected from 60 women in a period of 0 to 7 postpartum days. Women were healthy, normal weight, around 23 years-old, mostly with vaginal delivery (Table 1). Samples were collected in a period of three months from 16 October 2017 to 29 January 2018 (Figure S1). Total RNA was extracted, and miR-146b-5p, miR148a-3p, miR155-5p, mir181a-5p, mir200a-3p were quantified as described in Materials and Methods. Results for mir181a-5p were discarded due to technical problems with the primer FmiR181 (Table S2), which amplified a spurious product in the qPCR reaction for the non-template control. The results for miR-146b-5p, miR148a-3p, miR155-5p, and mir200a-3p indicated that the relative expression for all of them was comparable, as shown by the one-way ANOVA statistical test of the relative expression units (2^−ΔCт^) (Pr(>F) = 0.7136) (Figure 1). There is a tendency of higher miRNAs relative expression at postpartum day-3 (Figure S2). We also evaluated whether there was an association between miRNA relative expression and mother age but found no association in our data (Figure S3).

### 3.2. Selected miRNAs Were Identified in Infant Milk Formulae

**Figure 2:**
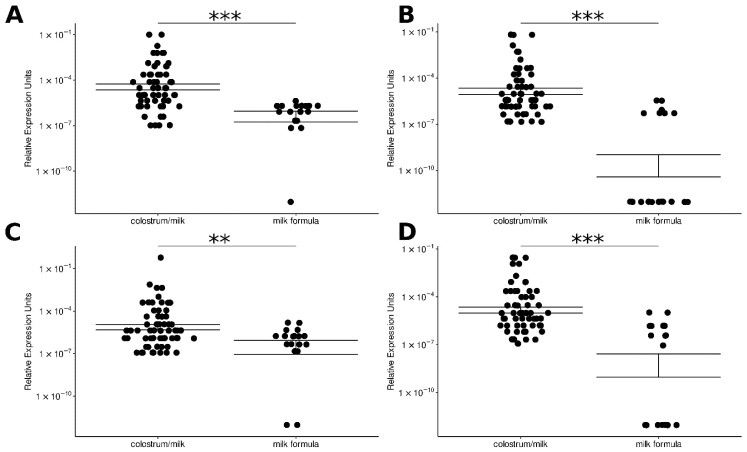
Comparative expression of selected hsa-miRs in colostrum/milk (n = 60) with respect to milk formula (n = 18). The graphics show the relative expression of each miRNA with respect to the internal control cel-miR-39. (**A**) hsa-miR-146, (**B**) hsa-miR-148, (**C**) hsa-miR-155, (**D**) hsa-miR-200. The Y-axis shows the relative expression of each miRNA, and the X-axis shows the type of sample. Each dot in the plot represents a sample. The double horizontal lines indicate the standard error of the mean. Mann-Whitney U (Wilcoxon rank-sum) test was made. ** indicates *p* < 0.01 and *** indicates *p* < 0.001.

Next, we studied the relative expression of miR-146b-5p, miR-148a-3p, miR-155-5p, mir-181a-5p, mir-200a-3p in six different infant milk formulae (IMF) available in the local market (Table S1). Total RNA was extracted after reconstituting the powder, as instructed by the manufacturer in triplicate. miR-146b-5p, miR-148a-3p, miR-155-5p, mir-181a-5p, and miR-200a-3p miRNAs were quantified as described in Materials and Methods. As mentioned before, the results for miR-181a-5p were discarded due to technical problems with the primer FmiR181 (Table S2). The results for miR-146b-5p, miR-148a-3p, miR-155-5p, and mir-200a-3p indicated that the relative expression of miR-146b-5p and miR-155-5p was higher in IMF than the expression of hsa-miR-148 and hsa-miR-200. In addition, the Mann-Whitney U (Wilcoxon rank-sum) test indicated that the relative expression of miRNAs was higher in the human milk samples in comparison to the IMF (*p* = 9.0 × 10^−7^ for miR-146; *p* = 1.1 × 10^−7^ for miR-148; *p* = 9.3 × 10^−3^ for miR-155, and *p* = 8.0 × 10^−6^ for mir-200) (Figure 2).

## 4. Discussion

miRNAs play an important role in the regulation of gene expression and contribute to the pathogenesis of complex diseases, such as IBD [28]. Several studies have revealed that levels of certain miRNAs are altered in IBD patients in comparison to healthy individuals, such as miR-124, miR-320, miR-21, miR-31, and miR-141 [29]. Further, work done on Crohn’s disease suggests that dysregulation of miRNAs at the level of the intestinal mucosa may play an important role in the early stages of the disease [30].

Studies in human milk (HM) have shown evidence of the presence of miRNAs, where they remain stable, even under very low pH conditions. This supports the notion that miRNAs can remain stable in the acidic conditions of the gastrointestinal tract and consequently, are able to be absorbed in the gut. Many miRNAs present in HM have been described, some with unknown function, but others with immunoregulatory function. Among the miRNAs found in a study of HM using massive sequencing, miR-146b-5p, miR-200a-3p, and miR-148-3p which were measured in our work, were found among the 10 most abundant [31]. A similar expression of miR-148a-3p, miR-146b-5p, and miR-200a/c-3p has been reported in different studies discussed in a recent systematic review, indistinctly of factors like the fraction of HM, lactational age, and health status of the mother and her offspring [32].

The miRNA expression after birth has been monitored. The expression of miRNA-146b, analyzed in the lipid and skimmed fraction of HM, was reported at relatively stable levels from the first to the second month after delivery [15]. In another work evaluating a different group of miRNAs, the expression of miR-146b-5p is reported stable in samples obtained during two, four, and six months after birth [33]. On the other hand, miR-148a suffered a slight decline in its expression in late lactation. Additionally, a differential expression was found between women with normal weight and women who are overweight or obese. A decrease of 30% was registered in obesity with respect to the healthy group during the first month of lactation. This decrease was not observed two months later, where significant differences in the expression of this miRNA among the groups were not found [34]. However, in another report, differences in the expression of miR-148 in skim milk (0 to 1 month postpartum) were not observed [35]. In another study, the presence of miR-200a-3p was reported consistently and highly expressed in all human milk samples evaluated [36]. Regarding variations in miRNA expression during the day, miR-146b has shown little variation in its expression [15].

It is noteworthy that studied miRNAs could be found in IMF despite the processing involved in their production, however, similar results, yielding lower miRNA in IMF with respect to HCM have been previously reported [7,37], despite the fact that miRNAs are known to be stable and resistant to different adverse conditions, like abrupt temperature and pH changes and endonuclease activity [31,38]. It is also important to remark that HCM samples used in this study were collected in late 2017 and early 2018, so they had been stored for at least three years at −70 °C, while the IMF were prepared and processed immediately after purchase. To this point, no reports have been found discussing the stability of miRNAs over prolonged periods of time, and although the long storage time of the samples could be a limitation of this work, it is interesting to note that there is a higher expression of the miRNAs studied in HCM, despite the age of the samples. Moreover, consistent with these results, another study focused on miRNA presence in infant gastric content and found that miRNA profile of gastric content of formula-fed infants was lower than that of breastfed infants, nevertheless, their presence indicates miRNA stability and transfer [39]. The limitations of this work are the small number of different immunoregulatory miRNAs and IMF that were monitored.

## 5. Conclusions

The relative expression of the four immunoregulatory miRNAs analyzed in this study is comparable in all the human colostrum/milk samples analyzed. The same miRNAs are not abundantly expressed in the infant milk formulae studied in this work. The expression of the immunomodulatory miRNAs is higher in human colostrum/milk than the expression in infant milk formulae. This observation supports the importance of the human breastfeeding practice. For instance, further studies analyzing human milk miRNA profile characterization by high-throughput sequencing could be useful. Human milk miRNA expression could be related with different factors, like breastfeeding period, mother’s age and weight, for this reason, appropriate studies are needed. Moreover, it is an important matter to compare human miRNA profile with different health conditions present in pregnancy, e.g., obesity, metabolic syndrome, and gestational diabetes.

## Figures and Tables

**Table 1:** Data for 60 participant mothers in the study.

**Anthropometric Data**		**Mean**	**SD**
	Age (years)	22.93	±4.95
	Height (m) ^α^	1.57	±0.07
	Weight (Kg) ^β^	56.48	±10.01
	BMI ^γ^	23.22	±3.74
**Type of delivery**		**n**	**%**
	Elective caesarean	4	6.67
	Non elective caesarean	13	21.67
	Vaginal	43	71.66
**Parity**		**n**	**%**
	Primiparous ^δ^	16	27.12
	Multiparous ^δ^	43	72.88
**Milk extraction**		n	%
	Manual ^ε^	34	82.93
	Pump ^ε^	7	17.07
**Socioeconomic data**		**n**	**%**
Residence	Mexico City (19°25′10″ N 99°08′44″ O)	15	25.00
	Estado de México (19°21′15″ N 99°37′51″ O)	36	60.00
	Guerrero State (17°36′47″ N 99°57′00″ O)	2	3.33
	Hidalgo State (20°28′42″ N 98°51′49″ O)	1	1.67
	Oaxaca State (16°53′53″ N 96°24′51″ O)	3	5.00
	Puebla State (19°00′13″ N 97°53′18″ O)	2	3.33
	Veracruz State (19°26′05″ N 96°22′59″ O)	1	1.67
Activity	Housewife ^δ^	52	88.14
	Student ^δ^	2	3.39
	General employee ^δ^	5	8.47
Education	Primary school (6 years)	20	33.33%
	Secondary school (3 years)	36	60.00%
	High school (3 years)	1	1.67%
	University school (4–5 years)	3	5.00%

^α^ 55 mothers; ^β^ 50 mothers; ^γ^ 48 mothers; ^δ^ 59 mothers; ^ε^ 41 mothers. BMI = weight (kg)/[height (m)]^2^, n, indicates number of data for the category. Anthropometric data correspond to the start of pregnancy.

## Data Availability

Not applicable.

## Supplementary Materials

The following are available online at https://www.mdpi.com/article/10.3390/biotech11020011/s1, Figure S1. Time frame graphic for sample procurement in the study, Figure S2. Expression of selected hsa-miRs in colostrum/milk at different time, Figure S3. Expression of miRNAs in colostrum/milk v.s. the age of mothers. Table S1. Nutrimental information of Infant Milk Formulae, and Table S2. Primer sequences used in this study.
