# Supplementary material for: Detection and Quantification of Immunoregulatory miRNAs in Human Milk and Infant Milk Formula
Source: BioTech (Basel). 2022 Apr 20;11(2):11. doi: 10.3390/biotech11020011 (PMC9264398; doi:10.3390/biotech11020011)
Supplement: Supplementary file 1 [file biotech-11-00011-s001.zip › biotech-1534795-supplementary.pdf]

BioTech

Detection and Quantification of immunoregulatory miRNAs in Human Milk and Infant Milk Formula

Juan Manuel Vélez-Ixta, Tizziani Benítez-Guerrero, Arlene Aguilera-Hernández, Helga Martínez-Corona, Karina Corona-Cervantes, Carmen Josefina Juárez-Castelán, Martín Noé Rangel-Calvillo, and Jaime García-Mena

Supplementary Material

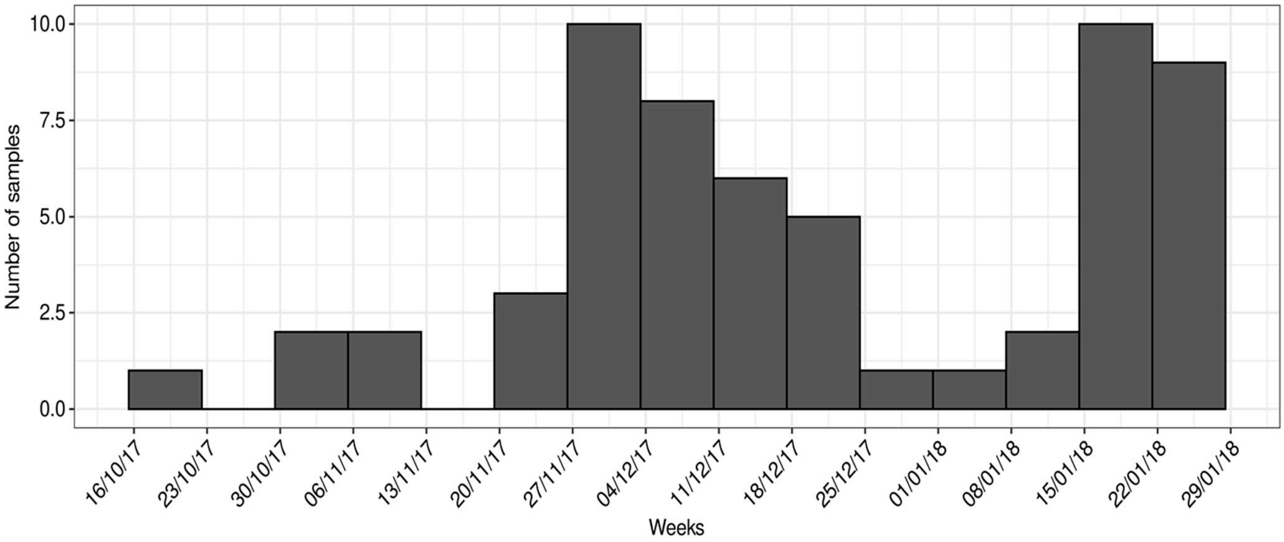

Figure S1. Time frame graphic for sample procurement in the study. The 60 samples were collected in approximately three months, between October 16th, 2017 and January 29th, 2018. The y-axis shows the number of samples, and the x-axis shows dates and weeks.

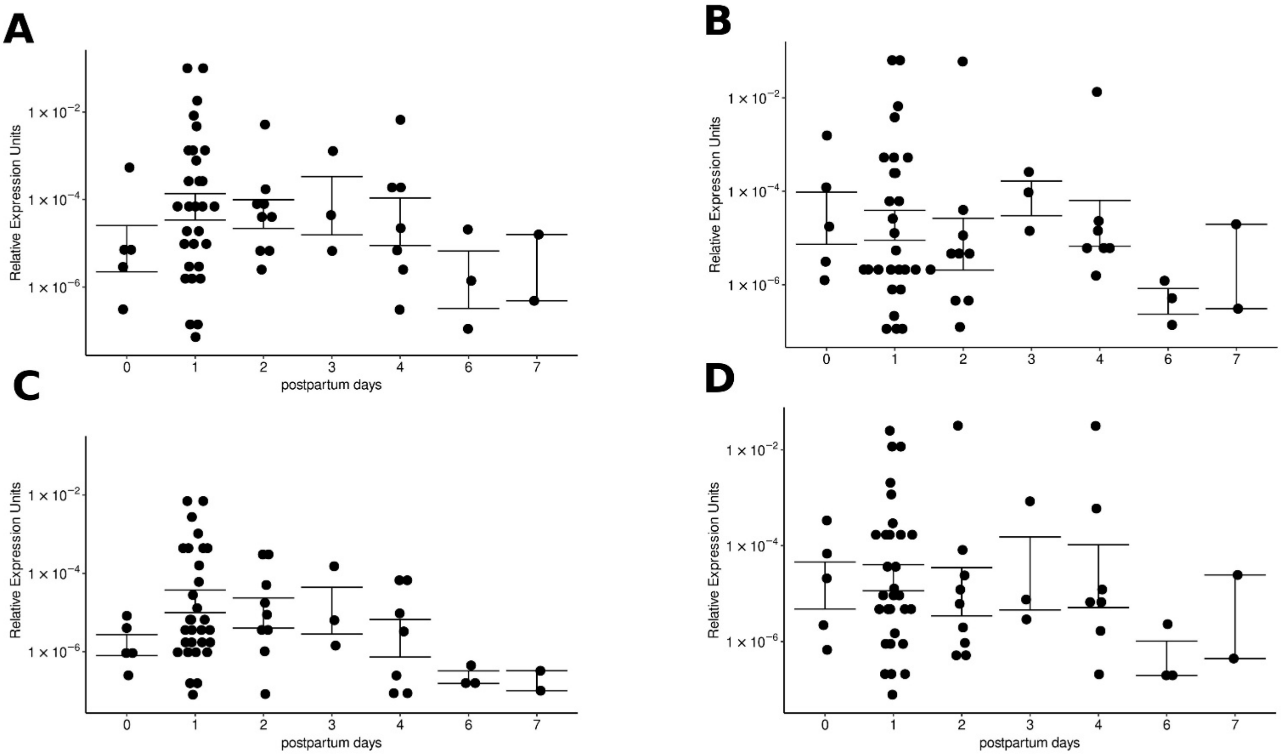

Figure S2. Expression of selected hsa-miRs in colostrum/milk at different time. a) hsa-miR-146, b) hsa-miR-148, c) hsa-miR-155, d) hsa-miR-200. The Y-axis shows the relative expression of each miRNA, and the X-axis shows the post-partum days. Each dot in the plot represents a sample. Day 0 (5), day 1 (30), day 2 (9), day 3 (3), day 4 (7), day 6 (3), day 7 (2). The double horizontal lines indicate the standard error of the mean. Kruskal-Wallis statistical test was made.

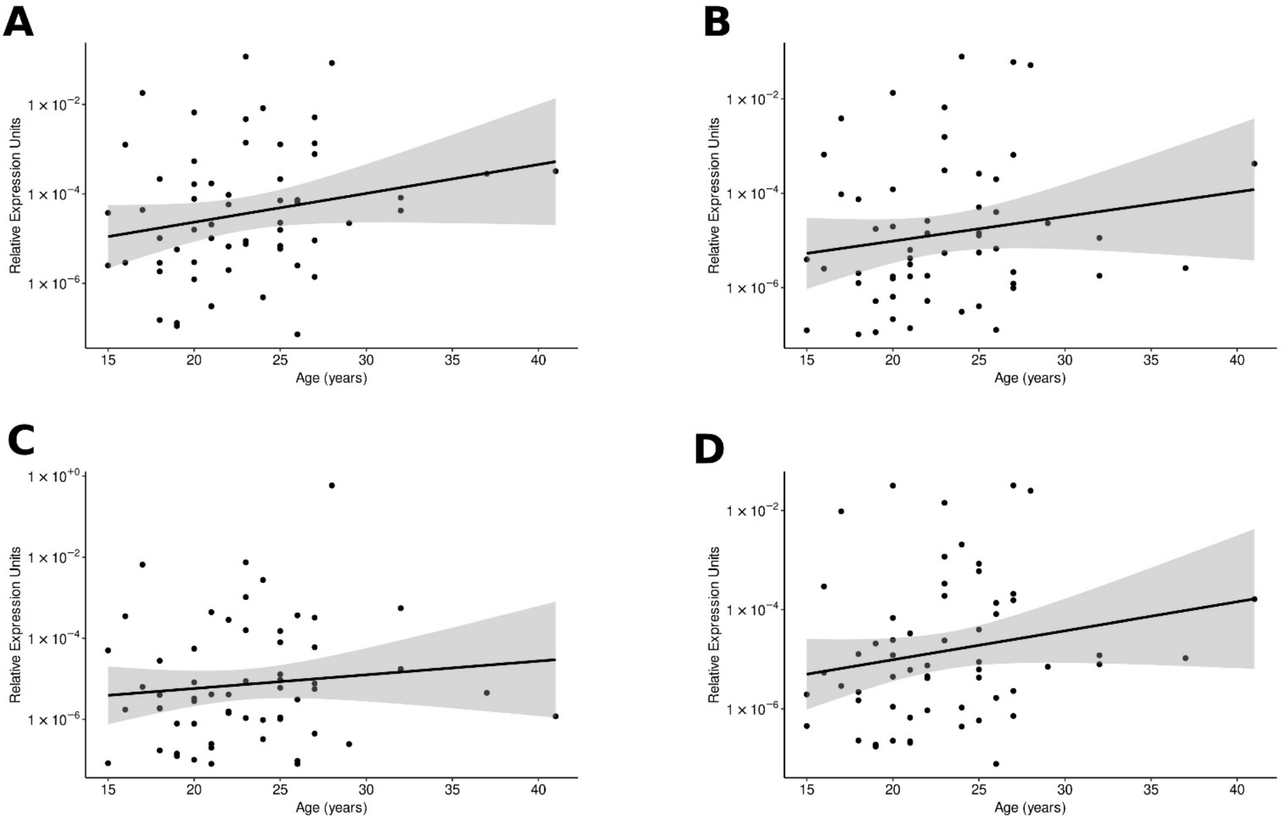

Figure S3. Expression of miRNAs in colostrum milk v.s. the age of mothers. a) hsa-miR-146 (*p*-value = 0.6224, R-Sq[adj] = -0.01319), b) hsa-miR-148 (*p*-value = 0.3396, R-Sq[adj] = -0.001289), c) hsa-miR-155 (*p*-value = 0.3112, R-Sq[adj] = 0.0007369), d) hsa-miR-200 (*p*-value = 0.6842, R-Sq[adj] = -0.01457). The Y-axis shows the relative expression of each miRNA, and the X-axis shows the age. Each dot represents a sample, horizontal line represents the linear model, and shaded areas represents the 95% confidence level interval for predictions within the linear model.

Table S1. Nutrimental information of Infant Milk Formulae

ID Formula[1]	Energetic content /100g/kcal	Carbohydrates/100g	Proteins/100g	Total fat/100g	Minerals/100g	Vitamins/100g	Other
1	506	57.8	11.3	25.5	Sodium 150 mg	Vitamin A 450 µg	Inositol 45 mg
					Potassium 470 mg	Thiamine B1 400 µg	Taurine 44 mg
					Chlorine 300 mg	Riboflavin B2 800 µg	L-carnitine 8 mg
					Calcium 380 mg	Pyridoxine B6 300 µg	
					Phosphorus 230 mg	Cyanocobalamin B12 1.5 µg	
					Magnesium 45 mg	Vitamin C 60 mg	
					Iron 6 mg	Vitamin D3 7.5 µg	
					Zinc 4.5 mg	Vitamin E 12.8 mg	
					Iodine 65 µg	Vitamin K1 30 µg	
					Copper 400 µg	Niacin B3 4.5 mg	
					Manganese 35 µg	Coline 100 mg	
					Selenium 10 µg	Pantothenic Acid 2.4 mg	
2	519	58.6	9.6	27.3	Sodium 180 µg	Vitamin E 7.5 µg	Inositol 70 mg
					Calcium 325 µg	Vitamin C 80 µg	Taurine 35 mg
					Phosphorus 180 µg	Pantothenic Acid 5 mg	Nucleotides 12 mg
					Iron 6,2 µg	Niacin B3 4 mg	Carnitine 7.8 mg
					Magnesium 40 µg	Vitamin A 450 µg	
					Zinc 5,3 µg	Thiamine B1 550 µg	
					Potassium 640 µg	Riboflavin B2 800 µg	
					Chlorides 360 µg	Pyridoxine B6 330 µg	
					Copper 400 µg	Folic Acid 60 µg	
					Selenium 15 µg	Cyanocobalamin B12 1,4 µg	
					Iodine 115 µg	Vitamin D 7.5	
					Manganese 90 µg	Vitamin K1 40	
						Biotin 13	
						Coline 100 mg	
3	511	59.35	9.72	26.08	Sodium 200 mg	Vitamin E 8.4 mg	Inositol 32 mg
					Potassium 580 mg	Vitamin C 69 mg	Taurine 30 mg
					Chlorides 385 mg	Pantothenic Acid 4.8	Nucleotides 15 mg

					Calcium 347 mg	Vitamin A 510 µg	Carnitine 13 mg
					Phosphorus 200 mg	Thiamine B1 500 µg	DHA 57 mg
					Magnesium 52 mg	Pyridoxine 360 µg	ARA 57 mg
					Iron 5.3 mg	Folic acid 81 µg	
					Zinc 5 mg	Vitamin d 6.8 µg	
					Copper 440 µg	Vitamin K1 40 µg	
					Manganese 95 µg	Biotin 11 µg	
					Iodine 70 µg	Riboflavin B2 1.25 mg	
					Selenium 16 µg	Cyanocobalamin 1.1 µg	
						Coline 56 mg	
4	478	54	9.2	25	Sodium 159 mg	Vitamin A 430 ug	Inositol 22 mg
					Potassium 630 mg	Vitamin D 6.3 µg	L-carnitine 7.2 mg
					Chlorine 410 mg	Vitamin C 97 mg	Taurine 29 mg
					Calcium 490 mg	Thiamine B1 370 µg	
					Phosphorus 290 mg	Riboflavin B2 870 µg	
					Magnesium 39 mg	Niacin B3 5700 µg	
					Iron 5.8 mg	Pyridoxine 320 ug	
					Iodine 70 µg	Folic acid 63 ug	
					Copper 300 µg	Pantothenic Acid 2800 µg	
					Zinc 3.1 mg	Cyanocobalamin 2.1 µg	
					Manganese 24 µg	Vitamin K1 68 µg	
					Selenium 8.7 µg	Biotin 13.5 µg	
						Vitamin E 5.8 µg	
						Coline 116 mg	
5	419	56.82	11.36	26.51	Sodium 171.59 mg	Vitamin A 492.42 µg	Taurine 37.88 mg
					Chlorine 306.29 mg	Vitamin C 75.76 mg	L-carnitine 8.33 mg
					Potassium 469.70 mg	Vitamin D 378.79 µg	Inositol 76.76 mg
					Calcium 416.67 mg	Vitamin K1 37.88 µg	DHA 49.24 mg
					Phosphorus 242.42 mg	Vitamin E	ARA 49.24 mg
					Magnesium 41.67 mg	Thiamine B1 0.38 mg	*Bifidobacterium lactis*
					Zinc 6.06 mg	Riboflavin B2 0.76 mg	
					Copper 318.18 µg	Niacin B3 3.79 mg	
					Iron 7.58 mg	Vitamin B6 0.38 mg	
					Manganese 49.24 µg	Vitamin B12 1.52 ug	

					Selenium 4.96 µg	Pantothenic Acid 2.50 mg	
					Iodine 75.76 µg	Biotin 12.12 ug	
						Coline 76.76 mg	
6	504	54	11	27	Calcium 385 mg	Vitamin A 500 µg	Nucleotides 20.1 mg
					Phosphorus 212 mg	Vitamin D3 8.5 µg	Taurine 47 mg
					Sodium 175 mg	Vitamin E 8.3 mg	Myo-inositol 40 mg
					Iron 6.3 mg	Vitamin K1 38 µg	L-Carnitine 20 mg
					Copper 335 µg	Vitamin B1 430 µg	ARA 63 mg
					Potassium 560 mg	Vitamin B2 560 µg	DHA 63 mg
					Chlorine 330 mg	Niacin B3 4200 µg	Galacto-oligosaccharides 1.9 g
					Magnesium 44 mg	Vitamin B6 440 µg	2-Fucosyl-Lactose 0.19 g
					Zinc 3.5 mg	Folic acid 87 µg	
					Iodine 96 µg	Pantothenic Acid 5000 ug	
					Manganese 123 µg	Vitamin B12 2.8 µg	
					Selenium 19 µg	Biotin 20 µg	
						Vitamin C 100 mg	
						Coline 130 mg	

1: Dairy formula from pasteurized cow's milk

Table S2. Primer sequences used in this study

Name	RT-primer (5′ - 3′)	Name	qPCR-primer (5′ - 3′)
RTmiR146	GTC-GTA-TCC-AGT-GCA-GGG-TCC-GAG-GTA-TTC-GCA-CTG-GAT-ACG-ACC-AGC-CT	FmiR146	CTC-CGA-GTG-AGA-ACT-GAA-TTC-C
RTmiR148	GTC-GTA-TCC-AGT-GCA-GGG-TCC-GAG-GTA-TTC-GCA-CTG-GAT-ACG-ACA-CAA-AG	FmiR148	AAG-CTT-CAG-TGC-ACT-ACA-GAA-C
RTmiR155	GTC-GTA-TCC-AGT-GCA-GGG-TCC-GAG-GTA-TTC-GCA-CTG-GAT-ACG-ACA-ACC-CC	FmiR155	GCG-TCT-CCT-TAA-TGC-TAA-TCG-TG
RTmiR181	GTC-GTA-TCC-AGT-GCA-GGG-TCC-GAG-GTA-TTC-GCA-CTG-GAT-ACG-ACA-CTC-AC	FmiR181	TAC-TGA-ACA-TTC-AAC-GCT-GTC-G
RTmiR200	GTC-GTA-TCC-AGT-GCA-GGG-TCC-GAG-GTA-TTC-GCA-CTG-GAT-ACG-ACA-CAT-CG	FmiR200	GGC-AGT-AAC-ACT-GTC-TGG-TAA-C
RTcel39	GTC-GTA-TCC-AGT-GCA-GGG-TCC-GAG-GTA-TTC-GCA-CTG-GAT-ACG-ACC-AAG-CT	Fcel39	CTG-CGT-CAC-CGG-GTG-TAA-ATC-A
		URP	TAT-CCA-GTG-CAG-GGT-CCG-A

RT: Retrotranscription, qPCR: quantitative Polymerase Chain Reaction, URP: Universal Reverse Primer. All RT-primers are 50-mer, and all qPCR-primer are 22-mer except for FmiR155 (23-mer), and URP (19-mer). The name of the primer indicates the hsa-miR target.

--end-of-file--
